# Distributed safe formation tracking control of multiquadcopter systems using barrier Lyapunov function

**DOI:** 10.3389/frobt.2024.1370104

**Published:** 2024-07-15

**Authors:** Nargess Sadeghzadeh-Nokhodberiz, Mohammad Reza Sadeghi, Rohollah Barzamini, Allahyar Montazeri

**Affiliations:** ^1^ Department of Control Engineering, Qom University of Technology, Qom, Iran; ^2^ Department of Electrical Engineering, Islamic Azad University Tehran Central Branch, Tehran, Iran; ^3^ School of Engineering, Lancaster University, Lancaster, United Kingdom

**Keywords:** multiagent systems, formation control, intervehicle collision avoidance, barrier Lyapunov function (BLF), formation tracking control, backstepping controller

## Abstract

Coordinating the movements of a robotic fleet using consensus-based techniques is an important problem in achieving the desired goal of a specific task. Although most available techniques developed for consensus-based control ignore the collision of robots in the transient phase, they are either computationally expensive or cannot be applied in environments with dynamic obstacles. Therefore, we propose a new distributed collision-free formation tracking control scheme for multiquadcopter systems by exploiting the properties of the barrier Lyapunov function (BLF). Accordingly, the problem is formulated in a backstepping setting, and a distributed control law that guarantees collision-free formation tracking of the quads is derived. In other words, the problems of both tracking and interagent collision avoidance with a predefined accuracy are formulated using the proposed BLF for position subsystems, and the controllers are designed through augmentation of a quadratic Lyapunov function. Owing to the underactuated nature of the quadcopter system, virtual control inputs are considered for the translational (*x* and *y* axes) subsystems that are then used to generate the desired values for the roll and pitch angles for the attitude control subsystem. This provides a hierarchical controller structure for each quadcopter. The attitude controller is designed for each quadcopter locally by taking into account a predetermined error limit by another BLF. Finally, simulation results from the MATLAB-Simulink environment are provided to show the accuracy of the proposed method. A numerical comparison with an optimization-based technique is also provided to prove the superiority of the proposed method in terms of the computational cost, steady-state error, and response time.

## 1 Introduction

Quadcopters are one of the most important categories of multirotor drones and consist of four arms, four motors, and four propellers. The control and navigation of quadcopters in a single or cooperative form have been the subject of various studies to enhance their capabilities for various applications (Montazeri et al., 2021; Sadeghzadeh-Nokhodberiz et al., 2023, 2021). A multiquadcopter system is a form of multiagent system that is used in various extreme environment applications, including nuclear decommissioning (Martin et al., 2016; Allahyar and Koubaa, 2023), volcanology (James et al., 2020), wildfire monitoring (Julian and Kochenderfer, 2019), and underground mining (Neumann et al., 2014). A multiagent system consists of several interacting intelligent agents that can cooperate their movements, sensing, and computations to achieve a common goal. Multiquadcopter systems are ideal solutions for different challenges imposed by humans working in extreme environments (Burrell et al., 2018); for example, using multiple quadcopters improves the performances, time and energy efficiencies, coverage areas, and redundancies of multiple robots performing the same task (Mansfield and Montazeri, 2024).

One of the most important issues in controlling a multiagent system is formation control to achieve consensus. Formation control is an important consideration in coordinating the control of a group of unmanned robots or quadcopters in the present study. It is assumed that each drone can fly and share information with the other robots in its neighborhood. Formation control is used in many applications relevant to environmental monitoring, such as coverage, patrolling, autonomous exploration, search and rescue, source seeking, and boundary tracking (Mansfield and Montazeri, 2024). In Liu and Bucknall (2018), the problem of formation control and cooperative motion planning of multiple unmanned vehicles was investigated and various approaches were reviewed; this work provides good insights into the challenges and techniques available for cooperative path planning and formation control.

One of the most investigated techniques to address the formation control problem is consensus-based formation control (Peng et al., 2020; Patil and Shah, 2021). Consensus is a displacement-based control mechanism, meaning that the agents simply need to know the relative locations (displacements) of their neighbors in a local reference system linked to a global system to achieve the desired formation. Displacement-based formation control is typically divided into three primary strategies: virtual structure (VS), behavior based (BB), and leader-follower (LF). The basic idea of consensus is that each vehicle updates its information state based on the information states of its local (time-varying) neighbors such that the final information state of each vehicle converges to a common value. The main purpose of a distributed formation control technique is to derive appropriate control commands for each agent based on the information provided by the agents that are only in the neighborhood of that agent. Here, the aim is that the team of robots should maintain a specific geometric shape while closely tracking the desired trajectory defined for the leader in the LF configuration or virtual leader in the formation control setting (Can et al., 2022; Imran and Montazeri, 2022). In such scenarios, the desired trajectory of each robot in a robotic fleet is not defined separately; instead, the trajectory should be defined, for example, for the center of the quad formation shape, under the connectivity assumption of the system graph that all agents can coordinate with the leader. Although this is a fully decentralized configuration, less centralized scenarios have also been reported in literature (Lizzio et al., 2022), in which the navigation was carried out at the ground control station and the desired trajectory was then transmitted to each drone that communicates with the neighboring agents to share their position and run the distributed on-board control algorithm to attain the desired trajectories. We adopted one such configuration in our investigation in this work.

The basic form of a formation control algorithm does not take into account the possibility of agent collisions while the agents attempt to reach their intended positions. For this reason, formation approaches considering interagent collision and/or obstacle avoidance have been the subject of investigations by some researchers. A comprehensive review comparing various collision avoidance strategies for unmanned aerial vehicle (UAV) applications can be found in Yasin et al. (2020). In the context of consensus-based formation control, similar collision avoidance strategies were surveyed and discussed by Sadeghzadeh-Nokhodberiz et al. (2023). When two drones generate a formation, they may collide with each other and obstacles in the transient phase as well as when reaching their desired positions and orientations. The consensus-based collision-free methods reviewed in both Lizzio et al. (2022) and Sadeghzadeh-Nokhodberiz et al. (2023) can be grouped under four main categories: (i) optimization-based techniques, (ii) force-field or artificial potential field (APF) techniques, (iii) geometric approaches, and (iv) sense-and-avoid approaches. As reviewed in Sadeghzadeh-Nokhodberiz et al. (2023), each of these approaches has its advantages/disadvantages for application to real life. Operationally speaking, static situations wherein obstacles are known ahead of time or are picked up on by the entire formation are more suited for optimization-based techniques. Although the force-field and geometric approaches are more effective for handling dynamic settings, the former may result in local minima due to cancellation of several APFs. Furthermore, the computational burden of a geometric technique in a busy dynamic environment may be very high when computing the collision-free trajectories. In terms of the operational requirements, optimization-based techniques are typically utilized in situations when the swarm must adhere to a predetermined reference trajectory. Depending on the algorithm chosen, each drone in the swarm either has a preloaded path or is aware of all the other reference trajectories. Nonetheless, the force-field and geometric techniques typically depend more on relative sensing or interagent communications.

Among the APF approaches reported for collision-free formation control, the method proposed by Yan et al. (2017) is notable because it causes the control signal to be limited and affected by the type of the potential field. Liang et al. (2020) studied a network of swarm drones, in which they followed a collision-free path by considering the system uncertainty in the presence of network constraints; the APF method was adopted here to address possible collisions between the UAVs, leading to a limited control signal. An example of an optimization-based technique used to design a collision-free formation control was reported by Kuriki and Namerikawa (2015); here, the problem was studied through the design of a consensus-based model predictive controller (MPC) by assuming that each UAV was located in a safe space and that the control input was updated as needed. The asymptotic stability of the proposed control method was also studied in detail. However, this method relied on the linear model of the system where the control system fails if the communication with the leader fails. Moreover, the collision avoidance strategy was considered only in the vertical direction. Jin et al. (2021) proposed a new framework to address the formation control of multiple robots; here, two types of problems were studied, namely the performance issues as well as feasibility of implementing the constraints when their requirements were in the tracking errors and distances between the paths.

Recently, reinforcement learning (RL) and deep reinforcement learning (DRL) techniques have been proven to be effective for decision-making and operation of cooperative robots in complex environments under time-varying and uncertain conditions. For example, Mansfield and Montazeri (2024) reviewed different multiagent RL (MARL) techniques used as advanced tools in the design of optimal cooperative trajectories for multirobot systems in environmental monitoring applications by optimizing not only the individual rewards of each of the robots but also their collective reward; although the focus of this was control and not desired trajectory design for quads operating in uncertain and complex environments, it was assumed that the target trajectory of each robot was designed and made available using the techniques of Liu and Bucknall (2018) or Mansfield and Montazeri (2024). Further, as mentioned in Mansfield and Montazeri (2024), the RL technique can be used to avoid interagent collisions and obstacles.

The above works do not use the barrier Lyapunov function (BLF) as an effective tool for collision-free formation tracking of quadcopters. More recently, Sadeghzadeh-Nokhodberiz and Meskin (2023) presented the problem of consensus-based formation tracking of multiquadcopter systems using logarithmic BLFs; however, the problem of collision avoidance was not considered. Instead, the method involved the use of a centralized approach that was then transformed to distributed control using highpass consensus filters. Although the performance of the proposed distributed method asymptotically converged to that of the centralized one, the convergence time was rather large. Therefore, the problem of collision-free formation tracking control of multiquadcopter systems is derived from scratch in a distributed manner in the present work.

Generally, the BLF is used to prevent the states from violating the constraints. Therefore, the BLF can be used to ensure safety and collision avoidance while guaranteeing convergence with a predefined accuracy. The BLF is a positive-definite function that grows to infinity when its arguments approach certain limits. Kumar and Kumar (2022) discussed the three-dimensional trajectory tracking problem of an unmanned vehicle with restrictions on the flight path during operations; to ensure that the quadrotor followed the desired trajectory while satisfying the imposed motion constraints, a BLF approach was proposed. Moreover, a six-degrees-of-freedom dynamic model of the system was considered to achieve high-accuracy tracking performance; this controller could avoid singularities in the attitude subsystem. Tang et al. (2013) proposed a single-input single-output non-linear control system using the BLF to avoid deviating from the safety range. Tee and Ge (2011) presented a feedback control system design with constraints on the states. Chen et al. (2020) studied the problem of obstacle avoidance for a system with multiple agents avoiding obstacles in the environment; in this method, a hybrid decentralized monitoring controller that guarantees collision avoidance was proposed. The method is scalable and can be applied to general non-linear robot dynamics. Recently, advanced model-based and uncertain optimal control laws have been developed and implemented in real time for impaired UAVs (Ahmadi et al., 2023). Ganguly (2022) used the BLF technique to design a controller for an N-degrees-of-freedom Euler–Lagrange system and numerically evaluated its effectiveness; this method was recently used for multirobot applications for interagent collision avoidance and tracking using second-order kinematics in two-dimensional cases (Jin et al., 2021). It is worth mentioning that Khadhraoui et al. (2023) and Mughees and Ahmad (2023) used BLFs for single quadcopter systems, in addition to Sadeghzadeh-Nokhodberiz and Meskin (2023), who recently employed BLFs for formation tracking of multiquadcopter systems without considering the collision avoidance problem.

Based on the above literature, a decentralized (distributed) collision-free formation tracking control method is proposed in this work for cooperative control of quadcopter systems. The proposed method is used for interagent collision avoidance and trajectory tracking with a predefined accuracy. Compared to the aforementioned works, the proposed method has a lower computational burden, is easily scalable, and can be used in dynamic environments. Further, contrary to the APF approaches, the proposed method does not limit the control signal for collision avoidance. The major contribution of the present study is that the barrier Lyapunov method is used to derive a distributed collision-free formation tracking control in which both formation tracking and interagent collision avoidance are considered simultaneously. Accordingly, BLFs are first proposed for the position subsystems (*x*, *y*, and *z* axes) and controllers are designed by augmenting a quadratic Lyapunov function, leading to a backstepping procedure. Owing to the underactuated nature of the quadcopter system, virtual inputs are considered for the translational (*x* and *y* axes) subsystems that are then used to generate the desired values for the roll and pitch angles for the attitude control subsystem. This provides a hierarchical controller structure for each quadcopter.

The distributed formation tracking controller derived herein not only guarantees convergence of the formation tracking error with a predefined accuracy but also avoids interagent collisions during the transient responses of the formation. Thus, both collision avoidance and trajectory tracking with a predefined bound on the tracking error are achieved in a distributed manner. The novelty of this work is briefly summarized as follows:

• Formulating multiple problems, including trajectory tracking, formation tracking control, and interagent collision avoidance, of a multiquadcopter system using the proposed BLF.
• Deriving decentralized (distributed) hierarchical control laws for collision-free formation tracking control of the altitude as well as translational *x* and *y* axes subsystems using virtual inputs in a backstepping framework.
• Designing attitude control laws separately for each agent using desired signals generated via BLFs while considering a predefined accuracy.

The remainder of this article is organized as follows. Section 2 details the problem formulation and preliminaries. Section 3 presents a decentralized collision-free formation tracking controller design for a multiquadcopter system using BLFs. Section 4 presents the simulation results, and Section 5 contains a summary of the conclusions.

## 2 Preliminaries and problem formulation

This section presents some preliminaries on the required theoretical materials.

### 2.1 Graph theory

Consider the graph $G=\left\{V,\xi ,W\right\}$ containing N nodes, where $V=\left\{1,2,\ldots ,N\right\}$ is the set of nodes and ξ is the set of all the edges of the graph. It is assumed that the edge $\left(i,j\right)$ between nodes i and j exists, where i and j are adjacent to each other, such that $\xi =\left(i,j\right)\in V\times V$ . If $\left(i,j\right)\in \xi \Leftrightarrow \left(j,i\right)\in \xi$ , then the graph is undirected. Matrix $A=\left[a_{ij}\right]$ is the adjacency matrix such that if there is a path from i to j in the system graph, then $a_{ij}=a_{ji}=1$ . A path from i to j is a sequence of distinct nodes starting at i and ending at j , such that each pair of consecutive nodes is adjacent. If there is a path from i to j , then the nodes are connected. If all the paths of a graph are connected, then the graph is connected. The degree matrix of a graph D is a diagonal matrix with elements $d_{i}$ that are equal to the set of neighboring nodes. $N_{i}=\left\{j\in V:\left(i,j\right)\in \xi \right\}$ , where $N_{i}$ is the set of neighbors surrounding i . The matrix L is the Laplacian matrix of the graph that is equal to L=D−A , and the sum of it rows is equal to zero (Hu et al., 2021).

### 2.2 Barrier Lyapunov theory

Consider the non-linear system given by Eq. (1) as follows:

$$\begin{array}{l} {\dot{x}}_{1}\left(t\right)=f_{1}\left(x_{1}\left(t\right)\right)+g_{1}\left(x_{1}\left(t\right)\right)x_{2}\left(t\right),\\ {\dot{x}}_{2}\left(t\right)=f_{2}\left(x_{1}\left(t\right),x_{2}\left(t\right)\right)+g_{1}\left(x_{1}\left(t\right),x_{2}\left(t\right)\right)u\left(t\right), \end{array}$$ (1)

where $x_{1}\left(t\right)\in R^{n_{1}}\text{ and }x_{2}\left(t\right)\in R^{n_{2}}$ are system states, $u\left(t\right)\in R^{n_{2}}$ is the system input, and vector functions $f_{1},f_{2},g_{1}$ , and $g_{2}$ are assumed to be smooth. The goal here is to design the control law $u\left(t\right)$ such that $x_{1}\left(t\right)$ follows the desired trajectory $x_{1d}\left(t\right)$ with a predefined accuracy. In other words, if $e_{1}\left(t\right):=x_{1}\left(t\right)-x_{1d}\left(t\right)$ , the control objective is to ensure that the tracking error remains within a compact set defined by $D_{1}=\left\{\left.e_{1}\left(t\right)\in R^{n_{1}}\right|d_{1}\left(t\right)=\left\|e_{1}\left(t\right)\right\|<\Omega_{1},t\geq 0\right\}$ , where $\Omega_{1}$ is a predefined positive scalar. Next, the idea of using the BLF in a backstepping procedure (Tee et al., 2009; Ngo et al., 2005; Tee et al., 2008) is extended to the vector form case. Therefore, assuming that the BLF $V_{1}\left(t\right)$ is defined as in Eq. (2), the Lyapunov candidate function $V\left(t\right)$ is defined by augmenting $V_{2}\left(t\right)$ to $V_{1}\left(t\right)$ as follows:

$$\begin{array}{l} V_{1}\left(t\right)=\frac{1}{2}\eta_{1}^{2}\left(t\right),\\ \;V\left(t\right)=V_{1}\left(t\right)+V_{2}\left(t\right), \end{array}$$ (2)

where $\eta_{1}\left(t\right)=\frac{\Omega_{1}d_{1}\left(t\right)}{\Omega_{1}-d_{1}\left(t\right)}$ and $V_{2}=\frac{1}{2}{z_{2}}^{T}z_{2}$ with $z_{2}\left(t\right)=x_{2}\left(t\right)-\alpha \left(t\right)$ is defined as an auxiliary tracking error for the virtual control input; $\alpha \left(t\right)$ is a stabilizing vector function that must be designed. According to Tee et al. (2009) and Lemma 1 therein, if the inequality $\dot{V}\left(t\right)\leq 0$ holds ∀t≥0 , it can be concluded that $e_{1}\left(t\right)\in D_{1}$ if $e_{1}\left(0\right)\in D_{1}$ .

### 2.3 Quadcopter model

Assume we have a group of quadcopters consisting of N agents communicating with each other. The dynamic of the attitude subsystem of the *i*th quadcopter (assuming a small Euler angle) for i=1,...,N can be written as follows:

$$\begin{array}{l} {\ddot{\phi }}_{i}\left(t\right)=\frac{I_{yy_{i}}-I_{zz_{i}}}{I_{xx_{i}}}{\dot{\theta }}_{i}\left(t\right){\dot{\psi }}_{i}\left(t\right)-I_{r_{i}}\Omega_{r_{i}}\frac{{\dot{\theta }}_{i}\left(t\right)}{I_{xx_{i}}}+\frac{u_{2i}\left(t\right)}{I_{xx_{i}}},\\ {\ddot{\theta }}_{i}\left(t\right)=\frac{I_{zz_{i}}-I_{xx_{i}}}{I_{yy_{i}}}{\dot{\phi }}_{i}\left(t\right){\dot{\psi }}_{i}\left(t\right)+I_{r_{i}}\Omega_{r_{i}}\frac{{\dot{\phi }}_{i}\left(t\right)}{I_{yy_{i}}}+\frac{u_{3i}\left(t\right)}{I_{yy_{i}}},\\ {\ddot{\psi }}_{i}\left(t\right)=\frac{I_{zz_{i}}-{I_{xx}}_{i}}{I_{yy_{i}}}{\dot{\phi }}_{i}\left(t\right){\dot{\theta }}_{i}\left(t\right)+\frac{u_{4i}\left(t\right)}{I_{zz_{i}}}. \end{array}$$ (3)

where the roll angle $\phi_{i}\left(t\right)$ , pitch angle $\theta_{i}\left(t\right)$ , and yaw angle $\psi_{i}\left(t\right)$ represent the rotations about the *x*, *y*, and *z* axes in the inertial frame, respectively. The input signals $u_{2i}\left(t\right)$ , $u_{3i}\left(t\right)$ , and $u_{4i}\left(t\right)$ represent torques in the corresponding directions for the *i*th quadcopter in the body frame. $I_{xx_{i}}$ , $I_{yy_{i}}$ , and $I_{zz_{i}}$ are the inertia tensors, and $I_{r_{i}}$ is the inertia of the propellers. Further, $\Omega_{r_{i}}$ describes the relative speed of the propeller.

The translational dynamics of the *i*th quadcopter can be presented as follows:

$$\begin{array}{l} {\ddot{x}}_{i}\left(t\right)=\frac{u_{1i}\left(t\right)}{m_{i}}\left(\cos\left(\psi_{i}\left(t\right)\right)\sin\left(\theta_{i}\left(t\right)\right)\cos\left(\phi_{i}\left(t\right)\right)+\sin\left(\psi_{i}\left(t\right)\right)\sin\left(\phi_{i}\left(t\right)\right)\right),\\ {\ddot{y}}_{i}\left(t\right)=\frac{u_{1i}\left(t\right)}{m_{i}}\left(\sin\left(\psi_{i}\left(t\right)\right)\sin\left(\theta_{i}\left(t\right)\right)\cos\left(\phi_{i}\left(t\right)\right)-\cos\left(\psi_{i}\left(t\right)\right)\sin\left(\phi_{i}\left(t\right)\right)\right),\\ {\ddot{z}}_{i}\left(t\right)=-g+\frac{u_{1i}\left(t\right)}{m_{i}}\cos\left(\phi_{i}\left(t\right)\right)\cos\left(\theta_{i}\left(t\right)\right), \end{array}$$ (4)

where ${\left[\begin{array}{ccc} x_{i}\left(t\right) & y_{i}\left(t\right) & z_{i}\left(t\right) \end{array}\right]}^{T}$ represents the position of *i*th quadcopter in the inertial frame, $u_{1i}\left(t\right)$ defines the main thrust created by the combined forces of the rotors, g is the gravitational constant, and $m_{i}$ refers to the mass of the *i*th quadcopter (Sadeghzadeh-Nokhodberiz et al., 2021).

The above dynamic system can be represented in the state-space form, and the system is divided into three subsystems for simplicity as altitude, translational, and attitude subsystems (Sadeghzadeh-Nokhodberiz et al., 2021).

The altitude subsystem can be decomposed as follows:

$$\begin{array}{l} {\dot{x}}_{1i}\left(t\right)=x_{2i}\left(t\right),\\ {\dot{x}}_{2i}\left(t\right)=-g+g_{2i}\left(t\right)u_{1i}\left(t\right), \end{array}$$ (5)

where $x_{1i}\left(t\right)\equiv z_{i}\left(t\right)$ and $x_{2i}\left(t\right)\equiv {\dot{z}}_{i}\left(t\right)$ refer to the altitude and velocity of the *i*th quadcopter in the *z* direction, respectively; $u_{1i}\left(t\right)$ is the control input indicating the thrust force applied to the *i*th quadcopter in the *z* direction; $g_{2i}\left(t\right)=\frac{1}{m_{i}}\cos\left(\theta_{i}\left(t\right)\right)\cos\left(\phi_{i}\left(t\right)\right)$ is an auxiliary variable defined to convert the last expression in Eq. (4) to a more compact form.

The translational subsystem is defined as follows:

$$\begin{array}{l} {\dot{x}}_{3i}\left(t\right)=x_{4i}\left(t\right),\\ {\dot{x}}_{4i}\left(t\right)=g_{4i}\left(t\right)u_{iv3}\left(t\right),\\ {\dot{x}}_{5i}\left(t\right)=x_{6i}\left(t\right),\\ {\dot{x}}_{6i}\left(t\right)=g_{6i}\left(t\right)u_{iv5}\left(t\right), \end{array}$$ (6)

where $x_{3i}\left(t\right)\equiv x_{i}\left(t\right)$ and $x_{4i}\left(t\right)\equiv {\dot{x}}_{i}\left(t\right)$ refer to the position and velocity of the *i*th quadcopter in the *x* direction, and $x_{5i}\left(t\right)\equiv y_{i}\left(t\right)$ and $x_{6i}\left(t\right)\equiv {\dot{y}}_{i}\left(t\right)$ refer to the position and velocity of the *i*th quadcopter in the *y* direction, respectively; $g_{4i}\left(t\right)=g_{6i}\left(t\right)=\frac{u_{1i}\left(t\right)}{m_{i}}$ are auxiliary variables defined to convert the last expression in Eq. (4) to a more compact form. Moreover, $u_{iv3}\left(t\right)$ and $u_{iv5}\left(t\right)$ are virtual controller inputs to enable control of the underactuated position subsystem and are defined as follows:

$$u_{iv3}\left(t\right)=\cos\left(\psi_{i}\left(t\right)\right)\sin\left(\theta_{i}\left(t\right)\right)\cos\left(\phi_{i}\left(t\right)\right)+\sin\left(\psi_{i}\left(t\right)\right)\cos\left(\phi_{i}\left(t\right)\right)$$ (7)

$$u_{iv5}\left(t\right)=\sin\left(\psi_{i}\left(t\right)\right)\sin\left(\theta_{i}\left(t\right)\right)\cos\left(\phi_{i}\left(t\right)\right)-\cos\left(\psi_{i}\left(t\right)\right)\sin\left(\phi_{i}\left(t\right)\right)$$ (8)

Finally, the attitude subsystem can be defined using Eq. (3) by assuming that $I_{r_{i}}$ is very small:

$$\begin{array}{l} {\dot{x}}_{7i}\left(t\right)=x_{8i}\left(t\right),\\ {\dot{x}}_{8i}\left(t\right)=f_{2i}\left(t\right)+G_{8i}u_{i}\left(t\right), \end{array}$$ (9)

where $x_{7i}\left(t\right)\equiv {\left[\begin{array}{ccc} \phi_{i}\left(t\right) & \theta_{i}\left(t\right) & \psi_{i} \end{array}\left(t\right)\right]}^{T}$ and $x_{8i}\left(t\right)\equiv {\left[\begin{array}{ccc} {\dot{\phi }}_{i}\left(t\right) & {\dot{\theta }}_{i}\left(t\right) & {\dot{\psi }}_{i} \end{array}\left(t\right)\right]}^{T}$ are the respective attitude and angular velocity vectors in the inertial frame; $u_{i}\left(t\right)={\left[\begin{array}{ccc} u_{2i}\left(t\right) & u_{3i}\left(t\right) & u_{4i} \end{array}\left(t\right)\right]}^{T}$ is the control input vector including the torques in the corresponding directions for the *i*th quadcopter in the body frame; $f_{2i}\left(t\right)={\left[\begin{array}{ccc} a_{1i}{\dot{\theta }}_{i}\left(t\right){\dot{\psi }}_{i}\left(t\right) & a_{3i}{\dot{\phi }}_{i}\left(t\right){\dot{\psi }}_{i}\left(t\right) & a_{5i}{\dot{\phi }}_{i}\left(t\right){\dot{\theta }}_{i}\left(t\right) \end{array}\right]}^{T}$ is an auxiliary vector with auxiliary variables defined by $a_{1i}=\frac{I_{yy_{i}}-I_{zz_{i}}}{I_{xx_{i}}}$ , $a_{3i}=\frac{I_{zz_{i}}-I_{xx_{i}}}{I_{yy_{i}}}$ , and $a_{5i}=\frac{I_{zz_{i}}-{I_{xx}}_{i}}{I_{yy_{i}}}$ ; $G_{8i}=\left[\begin{array}{ccc} b_{1i} & 0 & 0\\ 0 & b_{3i} & 0\\ 0 & 0 & b_{5i} \end{array}\right]$ is an auxiliary matrix with auxiliary variables $b_{1i}=\frac{1}{I_{xx_{i}}}$ , $b_{3i}=\frac{1}{I_{yy_{i}}}$ , and $b_{5i}=\frac{1}{I_{zz_{i}}}$ defined to ensure that the attitude dynamics defined in Eq. (3) are in a compact form.

Now, the following problems are considered in this work:

Problem 1 Formulating multiple problems, including trajectory tracking, formation tracking control, and interagent collision avoidance, for a multiquadcopter system such that the proposed barrier Lyapunov theory can be applied.

Problem 2 Deriving the decentralized (distributed) hierarchical control laws for collision-free formation tracking control for the altitude subsystem as well as translational *x* and *y* subsystems with virtual inputs in a backstepping framework.

Problem 3 Designing the attitude control laws separately for each agent using the desired signals generated via BLFs while considering a predefined accuracy.

## 3 Control objectives

Problem 1 is considered in this section. In this work, the goal is to design controllers $u_{1i}\left(t\right),...,u_{4i}\left(t\right)$ such that the control objectives are achieved. The control objective in this study is formation tracking control, which consists of two parts. First, each quadcopter should follow its specified desired trajectory with a predefined accuracy for position and orientation. Second, interagent collisions should be avoided based on specified bounds regarding how close the quadcopters can be.

### 3.1 Trajectory tracking error

Let $x_{1id}\left(t\right),i=1,2,\ldots ,N$ (where N indicates the number of quadcopters) be the desired altitude trajectory for the *i*th quadcopter that is continuous in time and has finite first- and second-order derivatives. Further, we define the altitude tracking error for the *i*th quadcopter as $e_{1i}\left(t\right)=x_{1i}\left(t\right)-x_{1id}\left(t\right)$ . The first control objective here is to ensure that the altitude of the *i*th quadcopter tracks the desired trajectory $x_{1id}\left(t\right)$ with a predefined accuracy; this can be formulated by ensuring that the altitude tracking error for the *i*th quadcopter remains with a compact set defined as follows:

$$D_{1ie}=\left\{\left.e_{1i}\left(t\right)\in R\right|d_{1ie}\left(t\right)=\left|e_{1i}\left(t\right)\right|<\Omega_{1idH},t\geq 0\right\},$$ (10)

where $\Omega_{1idH}$ is a positive scalar defined for the *i*th quadcopter with an upper bound for the tracking error.

Similar to the altitude, $x_{3id}\left(t\right)$ and $x_{5id}\left(t\right)$ are the desired translational trajectories for the *i*th quadcopter in the x and y directions, respectively. It is assumed that these desired trajectories are continuous in time and have limited first- and second-order derivatives. Further, $e_{3i}\left(t\right)=x_{3i}\left(t\right)-x_{3id}\left(t\right)$ and $e_{5i}\left(t\right)=x_{5i}\left(t\right)-x_{5id}\left(t\right)$ represent the tracking errors in the x and y directions for the *i*th quadcopter, respectively. The control objective here is to track the desired translational trajectories $x_{3id}\left(t\right)$ and $x_{5id}\left(t\right)$ with a predetermined accuracy; this can be formulated by ensuring that the translational tracking error for the *i*th quadcopter remains within a compact set defined as follows:

$$\begin{array}{l} D_{3ie}=\left\{\left.e_{3i}\left(t\right)\in R\right|d_{3ie}\left(t\right)=\left|e_{3i}\left(t\right)\right|<\Omega_{3idH},t\geq 0\right\},\\ D_{5ie}=\left\{\left.e_{5i}\left(t\right)\in R\right|d_{5ie}\left(t\right)=\left|e_{5i}\left(t\right)\right|<\Omega_{5idH},t\geq 0\right\}, \end{array}$$ (11)

where $\Omega_{3idH}$ and $\Omega_{5idH}$ are two separate positive scalars defined for the *i*th quadcopter in the *x* and *y* directions, respectively, with upper bounds for the tracking errors.

Finally, for the attitude subsystem, $x_{7id}\left(t\right)$ is considered as the desired trajectory vector for the *i*th quadcopter and assumed to be continuous in time with limited first- and second-order derivatives. Further, $e_{7i}\left(t\right)=x_{7i}\left(t\right)-x_{7id}\left(t\right)$ represents the attitude tracking error vector. The control objective here is to track the desired trajectory vector $x_{7id}\left(t\right)$ with a predefined accuracy; this can be formulated by ensuring that the attitude tracking error for the *i*th quadcopter remains within the compact set defined as follows:

$$D_{7ie}=\left\{\left.e_{7i}\left(t\right)\in R^{3}\right|d_{7ie}\left(t\right)=\left\|e_{7i}\left(t\right)\right\|<\Omega_{7idH},t\geq 0\right\},$$ (12)

where $\Omega_{7idH}$ is a predefined positive scalar with an upper bound for the tracking error, and $\left\|.\right\|$ is the 2-norm of the vector.

### 3.2 Collision avoidance and formation control

As introduced earlier, $x_{1i}\left(t\right)$ is the altitude of the *i*th quadcopter with $x_{1j}\left(t\right),j\in N_{i}$ being the altitudes of its neighboring agents. The goal here is that the distances of the real altitudes of each of the agents with their neighbors, i.e., $d_{1ij}\left(t\right)≜\left|x_{1i}\left(t\right)-x_{1j}\left(t\right)\right|,j\in N_{i}$ , will track the desired distances, i.e., $L_{1ij}\left(t\right)≜\left|x_{1id}\left(t\right)-x_{1jd}\left(t\right)\right|,j\in N_{i}$ , which are expressed by $d_{1ije}^{\prime }\left(t\right)≜\left|d_{1ij}\left(t\right)-L_{1ij}\left(t\right)\right|,j\in N_{i}$ with a predefined accuracy. Thus, formation control and interagent collision avoidance in the *z* direction are both guaranteed. This is achieved by ensuring that the error $d_{1ije}^{\prime }\left(t\right)$ for the *i*th quadcopter remains within the compact set defined as follows:

$$D_{1ijH}=\left\{\left.x_{1i}\left(t\right)\in R\right|d_{1ije}^{\prime }\left(t\right)<\Omega_{1ijH},j\in N_{i},t\geq 0\right\},$$ (13)

where $\Omega_{1ijH}$ is a positive predefined scalar with an upper bound for formation tracking and a collision avoidance bound.

The real distance of the *i*th quadcopter from its neighboring agents in the *x* direction is given by $d_{3ij}\left(t\right)≜\left|x_{3i}\left(t\right)-x_{3j}\left(t\right)\right|,j\in N_{i}$ , while the desired distance is represented by $L_{3ij}\left(t\right)≜\left|x_{3id}\left(t\right)-x_{3jd}\left(t\right)\right|,j\in N_{i}$ . Then, the goals of formation control and interagent collision avoidance in the *x* direction for the *i*th quadcopter are guaranteed with a predefined accuracy if $d_{3ije}^{\prime }\left(t\right)≜\left|d_{3ij}\left(t\right)-L_{3ij}\left(t\right)\right|,j\in N_{i}$ remains within the compact set defined as follows:

$$D_{3ijH}=\left\{\left.x_{3i}\left(t\right)\in R\right|d_{3ije}^{\prime }\left(t\right)<\Omega_{3ijH},j\in N_{i},t\geq 0\right\},$$ (14)

where $\Omega_{3ijH}$ is a positive predefined scalar with an upper bound for formation tracking and a collision avoidance bound.

Similarly, the real distance of the *i*th quadcopter from its neighboring agents in the *y* direction is given by $d_{5ij}\left(t\right)≜\left|x_{5i}\left(t\right)-x_{5j}\left(t\right)\right|,j\in N_{i}$ , while the desired distance is represented by $L_{5ij}\left(t\right)≜\left|x_{5id}\left(t\right)-x_{5jd}\left(t\right)\right|,j\in N_{i}$ . Then, the goals of formation control and interagent collision avoidance in the *y* direction for *i*th quadcopter are guaranteed with a predefined accuracy if $d_{5ije}^{\prime }\left(t\right)≜\left|d_{5ij}\left(t\right)-L_{5ij}\left(t\right)\right|,j\in N_{i}$ remains within the compact set defined as follows:

$$D_{5ijH}=\left\{\left.x_{5i}\left(t\right)\in R\right|d_{5ije}^{\prime }\left(t\right)<\Omega_{5ijH},j\in N_{i},t\geq 0\right\},$$ (15)

where $\Omega_{5ijH}$ is a positive predefined scalar with an upper bound for formation tracking and a collision avoidance bound.

Remark 1 It is worth noting that the problem considered here is neither LF control nor formation producing with/without a virtual leader. However, the shape of the formation is imposed on the method by appropriate design of the desired trajectory for each quadcopter.

## 4 Proposed distributed collision-free formation tracking control

Problem 2 is considered in this section, and the decentralized (distributed) hierarchical control laws for collision-free formation tracking control for the altitude and translational *x* and *y* subsystems with virtual inputs are designed in a backstepping framework for the multiquadcopter system. As mentioned earlier, because of the underactuated nature of the quadcopter system, a hierarchical procedure is employed. As the first step, the altitude controller is designed, and its result is used to design the controller for the translational subsystems along with virtual control inputs.

### 4.1 Altitude subsystem

Theorem 1 Assume that the altitude subsystem of the *i*th quadcopter in a fleet of N quadcopters is described by Eq. (5). Then, the altitude control input for the *i*th quadcopter $u_{1i}\left(t\right)$ can be designed as

$$u_{1i}\left(t\right)=g_{2i}^{-1}\left(t\right)\left[g+{\dot{\alpha }}_{1i}\left(t\right)-A_{1i}\left(t\right)-k_{2i}z_{2i}\left(t\right)\right];$$ (16)

where

$$\alpha_{1i}\left(t\right)={\dot{x}}_{1id}\left(t\right)+\frac{-k_{1i}e_{1i}^{2}\left(t\right)}{D_{1i}\left(t\right)e_{1i}\left(t\right)-2\sum_{j\in N_{i}}B_{1ij}\left(t\right)e_{1j}\left(t\right)},$$

$$A_{1i}\left(t\right)=D_{1i}\left(t\right)e_{1i}\left(t\right)-2\sum_{j\in N_{i}}B_{1ij}\left(t\right)e_{1j}\left(t\right),$$

$$D_{1i}\left(t\right)=B_{1i}\left(t\right)+2\sum_{j\in N_{i}}B_{1ij}\left(t\right),$$ (17)

with $B_{1i}=\frac{\Omega_{1idH}^{3}}{{\left(\Omega_{1idH}-d_{1ie}\left(t\right)\right)}^{3}}$ , $B_{1ij}=\frac{\Omega_{1ijH}^{3}}{{\left(\Omega_{1ijH}-{d^{\prime }}_{1ije}\left(t\right)\right)}^{3}}$ , $\Omega_{1ijH}>0,\Omega_{1idH}>0$ , and $k_{1i},k_{2i}>0$ . Then, the altitude tracking error and interagent collision avoidance conditions are guaranteed by remaining within the sets defined by Eqs. (10) and (13) if the quadcopter starts with the initial conditions such that the tracking errors remain within the same sets, i.e., $d_{1ie}\left(0\right)<\Omega_{1idH}$ and $d_{1ije}^{\prime }\left(0\right)<\Omega_{1ijH}$ , respectively.

Proof: We choose the following BLF candidate that contains the BLFs for each of the agents ( $V_{1ie}\left(t\right)$ ) as well as those related to the interagents ( $V_{1ij}\left(t\right)$ ):

$$V_{1i}\left(t\right)=\sum_{i=1}^{N}\left(V_{1ie}\left(t\right)+\sum_{j=1,j\neq i}^{N}a_{ij}V_{1ij}\left(t\right)\right),$$ (18)

where $V_{1ie}\left(t\right)=\frac{1}{2}\eta_{1ie}^{2}\left(t\right)$ and $V_{1ij}\left(t\right)=\frac{1}{2}\eta_{1ij}^{2}\left(t\right)$ , with $\eta_{1ie}\left(t\right)=\frac{\Omega_{1idH}d_{1ie}\left(t\right)}{\Omega_{1idH}-d_{1ie}\left(t\right)}$ and $\eta_{1ij}\left(t\right)=\frac{\Omega_{1ijH}d_{1ije}^{\prime }\left(t\right)}{\Omega_{1ijH}-d_{1ije}^{\prime }\left(t\right)}$ according to Eq. (2).

It is obvious from Eq. (18) that $V_{1i}$ is a positive-definite function. Therefore,

$$\begin{array}{ll} {\dot{V}}_{1i}\left(t\right) & =\sum_{i=1}^{N}\left({\dot{V}}_{1ie}\left(t\right)+\sum_{j=1,j\neq i}^{N}a_{ij}{\dot{V}}_{1ij}\left(t\right)\right)\\ & =\sum_{i=1}^{N}\left(\eta_{1ie}\left(t\right){\dot{\eta }}_{1ie}\left(t\right)+\sum_{j=1,j\neq i}^{N}a_{ij}\eta_{1ij}\left(t\right){\dot{\eta }}_{1ij}\left(t\right)\right), \end{array}$$ (19)

where ${\dot{V}}_{1ie}\left(t\right)=\eta_{1ie}\left(t\right){\dot{\eta }}_{1ie}\left(t\right)=\frac{\Omega_{1idH}^{3}}{{\left(\Omega_{1idH}-d_{1ie}\left(t\right)\right)}^{3}}d_{1ie}\left(t\right){\dot{d}}_{1ie}\left(t\right)$ ; further, by letting $B_{1i}\left(t\right)=\frac{\Omega_{1idH}^{3}}{{\left(\Omega_{1idH}-d_{1ie}\left(t\right)\right)}^{3}}$ , it can be concluded that ${\dot{V}}_{1ie}\left(t\right)=B_{1i}\left(t\right)d_{1ie}\left(t\right){\dot{d}}_{1ie}\left(t\right)$ . Since $d_{1ie}=\left|e_{1i}\right|$ , we have

$$\begin{array}{ll} {\dot{V}}_{1ie}\left(t\right) & =B_{1i}\left(t\right)\left|e_{1i}\left(t\right)\right|\frac{d}{dt}\left|e_{1i}\left(t\right)\right|\\ & =B_{1i}\left(t\right)\left|e_{1i}\left(t\right)\right|\text{sgn}\left(e_{1i}\left(t\right)\right){\dot{e}}_{1i}\left(t\right)\\ & =B_{1i}\left(t\right)e_{1i}\left(t\right){\dot{e}}_{1i}\left(t\right). \end{array}$$ (20)

Moreover, ${\dot{V}}_{1ij}\left(t\right)=\eta_{1ij}\left(t\right){\dot{\eta }}_{1ij}\left(t\right)=\frac{\Omega_{1ijH}^{3}}{{\left(\Omega_{1ijH}-{d^{\prime }}_{1ije}\left(t\right)\right)}^{3}}d_{1ije}^{\prime }\left(t\right){\dot{d}}_{1ije}^{\prime }\left(t\right)$ . Now, by letting $B_{1ij}\left(t\right)=\frac{\Omega_{1ijH}^{3}}{{\left(\Omega_{1ijH}-{d^{\prime }}_{1ije}\left(t\right)\right)}^{3}}$ , it is concluded that

$$\begin{array}{ll} {\dot{V}}_{ij}\left(t\right) & =B_{1ij}\left(t\right)d_{ije}^{\prime }\left(t\right){\dot{d}}_{ije}^{\prime }\left(t\right)\\ & =B_{1ij}\left(t\right)\left(e_{1i}\left(t\right)-e_{1j}\left(t\right)\right)\left({\dot{e}}_{1i}\left(t\right)-{\dot{e}}_{1j}\left(t\right)\right). \end{array}$$ (21)

Finally, by replacing Eqs. (20) and (21) in Eq. (19), we obtain

$${\dot{V}}_{1i}\left(t\right)=\sum_{i=1}^{N}\left(B_{1i}\left(t\right)e_{1i}\left(t\right){\dot{e}}_{1i}\left(t\right)+\sum_{j=1,j\neq i}^{N}a_{ij}B_{1ij}\left(t\right)\left(e_{1i}\left(t\right)-e_{1j}\left(t\right)\right)\left({\dot{e}}_{1i}\left(t\right)-{\dot{e}}_{1j}\left(t\right)\right)\right).$$ (22)

By rearranging Eq. (22), we have

$$\begin{array}{ll} {\dot{V}}_{1i}\left(t\right) & =\sum_{i=1}^{N}B_{1i}\left(t\right)e_{1i}\left(t\right){\dot{e}}_{1i}\left(t\right)+\sum_{i=1}^{N}\sum_{j=1}^{N}a_{ij}B_{1ij}\left(t\right)\left(e_{1i}\left(t\right)-e_{1j}\left(t\right)\right){\dot{e}}_{1i}\left(t\right)\\ & \;-\sum_{i=1}^{N}\sum_{j=1}^{N}a_{ij}B_{1ij}\left(t\right)\left(e_{1i}\left(t\right)-e_{1j}\left(t\right)\right){\dot{e}}_{1j}\left(t\right). \end{array}$$ (23)

Using the summation properties and the fact that $a_{ij}=a_{ji}$ and $B_{1ij}\left(t\right)=B_{1ji}\left(t\right)$ , it is concluded that $-\sum_{i=1}^{N}\sum_{j=1}^{N}a_{ij}B_{1ij}\left(t\right)\left(e_{1i}\left(t\right)-e_{1j}\left(t\right)\right){\dot{e}}_{1j}\left(t\right)=\sum_{i=1}^{N}\sum_{j=1}^{N}a_{ij}B_{1ij}\left(t\right)\left(e_{1i}\left(t\right)-e_{1j}\left(t\right)\right){\dot{e}}_{1i}\left(t\right)$ ; hence, Eq. (23) can be rewritten as

$${\dot{V}}_{1i}\left(t\right)=\sum_{i=1}^{N}\left(B_{1i}\left(t\right)e_{1i}\left(t\right)+2\sum_{j=1}^{N}a_{ij}B_{1ij}\left(t\right)\left(e_{1i}\left(t\right)-e_{1j}\left(t\right)\right)\right){\dot{e}}_{1i}\left(t\right).$$ (24)

Assuming that $z_{2i}\left(t\right)=x_{2i}\left(t\right)-\alpha_{1i}\left(t\right)$ , we have $x_{2i}\left(t\right)=z_{2i}\left(t\right)+\alpha_{1i}\left(t\right)$ . Since ${\dot{e}}_{1i}\left(t\right)=x_{2i}\left(t\right)-{\dot{x}}_{1id}\left(t\right)$ , the expression can be rewritten as ${\dot{e}}_{1i}\left(t\right)=z_{2i}\left(t\right)+\alpha_{1i}\left(t\right)-{\dot{x}}_{1id}\left(t\right)$ . Now, substituting this into Eq. (24), the stabilizing function $\alpha_{1i}$ is derived as in Eq. (17). Therefore, Eq. (24) can be rewritten as ${\dot{V}}_{1i}\left(t\right)=\sum_{i=1}^{N}\left[A_{1i}\left(t\right)z_{2i}-k_{1i}e_{1i}^{2}\left(t\right)\right]$ . By defining a backstepping-type Lyapunov function candidate and adding a quadratic function to $V_{1i}\left(t\right)$ , we have $V_{2i}\left(t\right)=V_{1i}\left(t\right)+\frac{1}{2}\sum_{i=1}^{N}z_{2i}^{2}\left(t\right)$ . Taking the derivative of the Lyapunov function gives

$$\begin{array}{ll} {\dot{V}}_{2i}\left(t\right) & ={\dot{V}}_{1i}\left(t\right)+\sum_{i=1}^{N}z_{2i}\left(t\right){\dot{z}}_{2i}\left(t\right)\\ & ={\dot{V}}_{1i}\left(t\right)+\sum_{i=1}^{N}z_{2i}\left(t\right)\left(-g+g_{4i}\left(t\right)u_{1i}\left(t\right)-{\dot{\alpha }}_{1i}\left(t\right)\right). \end{array}$$ (25)

Replacing $u_{1i}\left(t\right)$ from Eq. 16 into 25 gives

$$\begin{array}{ll} {\dot{V}}_{2i}\left(t\right) & =-\sum_{i=1}^{N}k_{1i}e_{1i}^{2}\left(t\right)+\sum_{i=1}^{N}A_{1i}\left(t\right)z_{2i}\left(t\right)\\ & \;+\sum_{i=1}^{N}z_{2i}\left(t\right)\left(-g-{\dot{\alpha }}_{1i}\left(t\right)+g+{\dot{\alpha }}_{1i}\left(t\right)-A_{1i}\left(t\right)-k_{2i}z_{2i}\left(t\right)\right)\\ & =-\sum_{i=1}^{N}k_{1i}z_{1i}^{2}\left(t\right)-\sum_{i=1}^{N}k_{2i}z_{2i}^{2}\left(t\right). \end{array}$$ (26)

Therefore, one can conclude from Eq. (26) that ${\dot{V}}_{2i}\left(t\right)<0$ , which completes the proof.

### 4.2 Translational subsystems

Herein, the virtual controllers for the translational subsystems presented in Eq. (6) for the x and y coordinates are formulated in accordance with Theorem 2.

Theorem 2 Assume that the translational subsystems of the *i*th quadcopter in a fleet of N quadcopters can be described by Eq. (6) in the *x* and *y* directions using the virtual control inputs defined in Eqs. (7) and (8). Then, the virtual control inputs $u_{iv3}\left(t\right)$ and $u_{iv5}\left(t\right)$ for the *i*th quadcopter can be designed as

$$\begin{array}{l} u_{iv3}\left(t\right)=g_{4i}^{-1}\left(t\right)\left[{\dot{\alpha }}_{3i}\left(t\right)-A_{3i}\left(t\right)-k_{4i}z_{4i}\left(t\right)\right],\\ u_{iv5}\left(t\right)=g_{6i}^{-1}\left[{\dot{\alpha }}_{5i}\left(t\right)-A_{5i}\left(t\right)-k_{6i}z_{6i}\left(t\right)\right], \end{array}$$ (27)

where

$$\begin{array}{l} \alpha_{3i}\left(t\right)={\dot{x}}_{3id}\left(t\right)+\frac{-k_{3i}e_{3i}^{2}\left(t\right)}{D_{3i}\left(t\right)e_{3i}\left(t\right)-2\sum_{j\in N_{i}}^{N}B_{3ij}\left(t\right)e_{3j}\left(t\right)},\\ A_{3i}\left(t\right)=D_{3i}\left(t\right)e_{3i}\left(t\right)-2\sum_{j\in N_{i}}^{N}B_{3ij}\left(t\right)e_{3j}\left(t\right),\\ D_{3i}\left(t\right)=B_{3i}\left(t\right)+2\sum_{j\in N_{i}}^{N}B_{3ij}\left(t\right), \end{array}$$ (28)

$$\begin{array}{l} \alpha_{5i}\left(t\right)={\dot{x}}_{5id}\left(t\right)+\frac{-k_{5i}e_{5i}^{2}\left(t\right)}{D_{5i}\left(t\right)e_{5i}\left(t\right)-2\sum_{j\in N_{i}}^{N}B_{5ij}\left(t\right)e_{5j}\left(t\right)},\\ A_{5i}\left(t\right)=D_{5i}\left(t\right)e_{5i}\left(t\right)-2\sum_{j\in N_{i}}^{N}B_{5ij}\left(t\right)e_{5j}\left(t\right),\\ D_{5i}\left(t\right)=B_{5i}\left(t\right)+2\sum_{j\in N_{i}}^{N}B_{5ij}\left(t\right), \end{array}$$

with $B_{3i}\left(t\right)=\frac{\Omega_{3idH}^{3}}{{\left(\Omega_{3idH}-d_{3ie}\left(t\right)\right)}^{3}},$ $B_{3ij}\left(t\right)=\frac{\Omega_{3ijH}^{3}}{{\left(\Omega_{3ijH}-{d^{\prime }}_{3ije}\left(t\right)\right)}^{3}},$ $B_{5i}\left(t\right)=\frac{\Omega_{5idH}^{3}}{{\left(\Omega_{5idH}-d_{5ie}\left(t\right)\right)}^{3}},$ $B_{5ij}\left(t\right)=\frac{\Omega_{5ijH}^{3}}{{\left(\Omega_{5ijH}-{d^{\prime }}_{5ije}\left(t\right)\right)}^{3}},$ $z_{4i}\left(t\right)=x_{4i}\left(t\right)-\alpha_{3i}\left(t\right),$ $z_{6i}\left(t\right)=x_{6i}\left(t\right)-\alpha_{5i}\left(t\right)$ , $k_{3i}>0,k_{4i}>0$ , $k_{5i}>0$ , $k_{6i}>0$ , $\Omega_{1ijH}>0,$ and $\Omega_{1idH}>0$ .

Then, the translational tracking error and interagent collision avoidance conditions are guaranteed by remaining within the sets defined by Eqs. (11), (14), and (15) if the quadcopter starts with the initial conditions such that the tracking errors remain within the same sets, i.e., $d_{3ie}\left(0\right)<\Omega_{3idH}$ , $d_{5ie}\left(0\right)<\Omega_{5idH}$ , $d_{3ije}^{\prime }\left(0\right)<\Omega_{3ijH}$ , and $d_{5ije}^{\prime }\left(0\right)<\Omega_{5ijH}$ , respectively.

Proof: A proof similar to that of Theorem 1 can be considered here and has been omitted for brevity.

## 5 Proposed attitude control system

Problem 3 is considered in this section, and a BLF-based controller is designed for the attitude subsystem with the dynamics presented in Eq. (9). First, according to Eqs. (7) and (8) as well as the virtual controllers designed for the translational subsystems in Eqs (27) and (28) the desired angles for the roll ( $\phi_{id}\left(t\right)$ ) and pitch ( $\theta_{di}\left(t\right)$ ) are computed in Eq. (29) as follows:

$$\begin{array}{l} \sin\left(\phi_{id}\left(t\right)\right)=u_{iv3}\left(t\right)\sin\left(\psi_{i}\left(t\right)\right)-u_{iv5}\left(t\right)\cos\left(\psi_{i}\left(t\right)\right)\\ \sin\left(\theta_{di}\left(t\right)\right)=\frac{u_{iv3}\left(t\right)\cos\left(\psi_{i}\left(t\right)\right)+u_{iv5}\left(t\right)\sin\left(\psi_{i}\left(t\right)\right)}{\cos\left(\phi_{id}\left(t\right)\right)} \end{array}$$ (29)

The desired yaw angle ( $\psi_{id}\left(t\right)$ ) can be set freely.

Theorem 3 Assume that the attitude subsystem of the *i*th quadcopter in a fleet of N quadcopters can be described by Eq. (9); then, the control input vector $u_{i}\left(t\right)$ for the *i*th quadcopter can be designed as

$$u_{i}\left(t\right)=G_{8i}^{-1}\left(-f_{2i}\left(t\right)+{\dot{\alpha }}_{7i}\left(t\right)-B_{7i}\left(t\right)e_{7i}\left(t\right)-k_{8i}z_{8i}\left(t\right)\right),$$ (30)

where $z_{8i}\left(t\right)=x_{8i}\left(t\right)-\alpha_{8i}\left(t\right)$ and $\alpha_{8i}\left(t\right)={\dot{x}}_{7id}-\left(k_{7i}e_{7i}\left(t\right)/B_{7i}\left(t\right)\right)$ , with $B_{7i}=\frac{\Omega_{7idH}^{3}}{{\left(\Omega_{7idH}-d_{7ie}\right)}^{3}}$ and $k_{7i}$ , $k_{8i}$ , and $\Omega_{7idH}$ being scalar positive constants.

Then, the attitude tracking error is guaranteed by remaining within the set defined in Eq. (12) if the quad starts from the initial conditions such that the tracking errors remain within the same sets, i.e., $d_{7ie}\left(0\right)=\left\|e_{7i}\left(0\right)\right\|<\Omega_{7idH}$ .

Proof: We consider the BLF $V_{7i}\left(t\right)=\frac{1}{2}\eta_{7ie}^{2}\left(t\right)$ with $\eta_{7ie}\left(t\right)=\frac{\Omega_{7idH}d_{7ie}\left(t\right)}{\Omega_{7idH}-d_{7ie}\left(t\right)}$ ; therefore,

$$\begin{array}{ll} {\dot{V}}_{7i}\left(t\right) & =\frac{\Omega_{7idH}^{3}}{{\left(\Omega_{7idH}-d_{7i}\left(t\right)\right)}^{3}}e_{7i}^{T}\left(t\right){\dot{e}}_{7i}\left(t\right)\\ & =B_{7i}\left(t\right)e_{7i}^{T}\left(t\right){\dot{e}}_{7i}\left(t\right). \end{array}$$ (31)

Since ${\dot{e}}_{7i}\left(t\right)=x_{8}\left(t\right)-{\dot{x}}_{7id}\left(t\right)=z_{8i}\left(t\right)+\alpha_{8i}\left(t\right)-{\dot{x}}_{7id}\left(t\right)$ , if we select $\alpha_{8i}\left(t\right)={\dot{x}}_{7id}-\left(k_{7i}e_{7i}\left(t\right)/B_{7i}\left(t\right)\right)$ ,we obtain ${\dot{V}}_{7i}\left(t\right)=B_{7i}\left(t\right)e_{7i}^{T}\left(t\right)z_{8i}\left(t\right)-k_{7i}e_{7i}^{T}\left(t\right)e_{7i}\left(t\right)$ . Now, a Lyapunov function is chosen by adding a quadratic function to $V_{7i}$ as follows:

$$V_{8i}\left(t\right)=V_{7i}\left(t\right)+\frac{1}{2}z_{8i}^{T}\left(t\right)z_{8i}\left(t\right).$$ (32)

Therefore, using Eq. (32) one can conclude Eq. (33) as follows:

$$\begin{array}{ll} {\dot{V}}_{8i}\left(t\right) & ={\dot{V}}_{7i}\left(t\right)+z_{8i}^{T}\left(t\right){\dot{z}}_{8i}\left(t\right)\\ & =B_{7i}\left(t\right)e_{7i}^{T}\left(t\right)z_{8i}\left(t\right)-k_{7i}e_{7i}^{T}\left(t\right)e_{7i}\left(t\right)\\ & \;+z_{8i}^{T}\left(t\right)\left({\dot{x}}_{8i}\left(t\right)-{\dot{\alpha }}_{8i}\left(t\right)\right). \end{array}$$ (33)

Now, according to Eq. (9), by selecting $u_{i}\left(t\right)$ as Eq. (30) and using Eq. (31) it is concluded that ${\dot{V}}_{8i}\left(t\right)=-k_{7i}e_{7i}^{T}\left(t\right)e_{7i}\left(t\right)-k_{8i}z_{8i}^{T}\left(t\right)z_{8i}\left(t\right)<0$ . Therefore, the attitude tracking objective in Eq. (12) is satisfied if $d_{7ie}\left(0\right)=\left\|e_{7i}\left(0\right)\right\|<\Omega_{7idH}$ , hence completing the proof.

**FIGURE 1:**
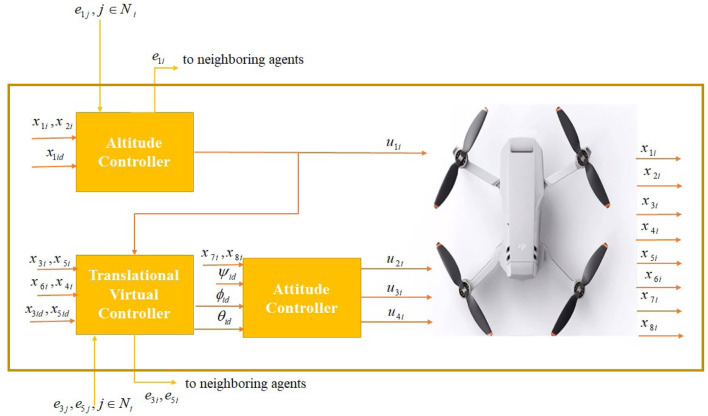
General framework of the proposed controller for the *i*th agent.

Figure 1 depicts the general structure of the proposed controller for the *i*th agent. The overall quadcopter system has three subsystems. The design of $u_{1i}\left(t\right)$ starts from the altitude subsystem. Then, this controller is used to design the virtual controllers in the translational subsystems. Finally, the control inputs of the attitude subsystem are designed to meet the desired control objectives. Owing to the fact that the graph topology of the quadcopter system is connected, the neighboring information is used to achieve safety, collision avoidance, and stability.

## 6 Simulation results

**FIGURE 2:**
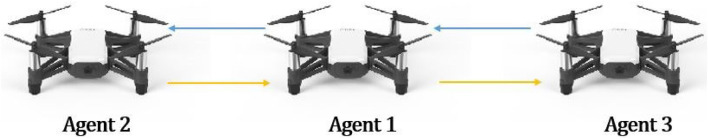
Interconnections between the agents in the simulation.

In this section, simulation results are provided to demonstrate the efficiency of the proposed method. Figure 2 depicts the interconnection of three quadcopters considered for the simulation.

The initial conditions are considered as follows: $x_{11}\left(0\right)=0,x_{12}\left(0\right)=0.5,x_{13}\left(0\right)=1$ , $x_{21}\left(0\right)=x_{22}\left(0\right)=x_{23}\left(0\right)=-0.08$ , $x_{31}\left(0\right)=x_{32}\left(0\right)=x_{33}\left(0\right)=0$ , $x_{41}\left(0\right)=x_{42}\left(0\right)=x_{43}\left(0\right)=1.5$ , $x_{51}\left(0\right)=x_{52}\left(0\right)=x_{53}\left(0\right)=3.9$ , $x_{61}\left(0\right)=x_{62}\left(0\right)=x_{63}\left(0\right)=0$ , $x_{7i}\left(0\right)=\left[\begin{array}{ccc} 0 & 0 & 0 \end{array}\right],i=1,2,3$ , and $x_{8i}\left(0\right)=\left[\begin{array}{ccc} 0 & 0 & 0 \end{array}\right],i=1,2,3$ . The physical parameters of the quadcopters are as follows: $m_{i}=1.47kg$ , $I_{xxi}=I_{yyi}=0.01152\;kgm^{2}$ , $I_{zzi}=0.0218\;kgm^{2}$ , and $L_{i}=0.28$ , i=1,2,3 . The reference trajectory for the movement of the quadcopters is given in Eq. (34) as follows:

$$\begin{array}{l} z_{1d}\left(t\right)=-0.1t,z_{2d}\left(t\right)=-0.1t+0.5,z_{3d}\left(t\right)=-0.1t+1\\ x_{1d}\left(t\right)=x_{2d}\left(t\right)=x_{3d}\left(t\right)=4\sin\left(0.5t\right)\\ y_{1d}\left(t\right)=y_{2d}\left(t\right)=y_{3d}\left(t\right)=4\cos\left(0.5t\right). \end{array}$$ (34)

As mentioned previously, the values $\Omega_{1idH}>0$ to $\Omega_{1=7idH}>0$ are the upper limits for distance tracking errors $d_{1ie}$ to $d_{7ie}$ , while $\Omega_{1ijH}>0$ , $\Omega_{3ijH}>0$ , and $\Omega_{5ijH}>0$ are the respective upper limits for the distance tracking errors $d_{1ije}^{\prime }$ , $d_{3ije}^{\prime }$ , and $d_{5ije}^{\prime }$ . These two sets of parameters determine the safe sets for the movements of the quadcopters. If these values are selected to be large, although the safety set will be larger, it may cause problems for the system in terms of safety as a wider range of errors would be considered acceptable. If these values are too small, then the safe set will be too small and forces the selection of the initial values to be very close to the real ones, which is unrealistic and may force the algorithm to be very sensitive to small deviations of the errors. Therefore, the selection of these two sets of parameters is very important. In the simulations, they are selected as follows: $\Omega_{7idH}=1.5$ , $\Omega_{1idH}=\Omega_{1ijH}=0.1$ , $\Omega_{3idH}=\Omega_{3ijH}=0.49,\text{and }\Omega_{5idH}=\Omega_{5ijH}=0.25$ . The simulation results are as follows.

**FIGURE 3:**
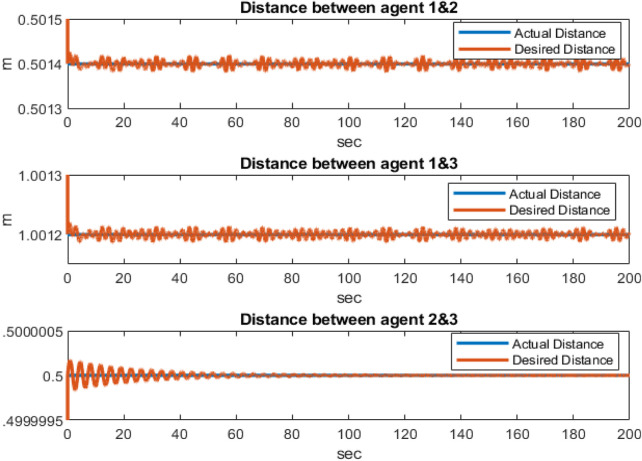
Distances between the agents in the simulation.

**FIGURE 4:**
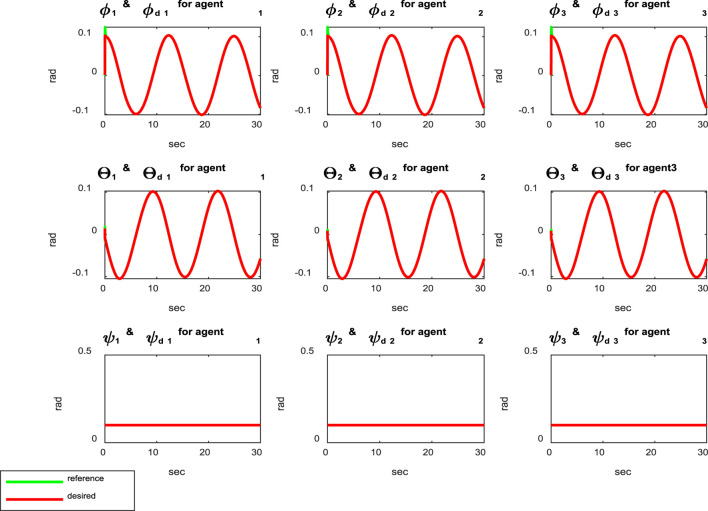
Comparison of the reference and actual trajectory states for the attitude subsystem.

**FIGURE 5:**
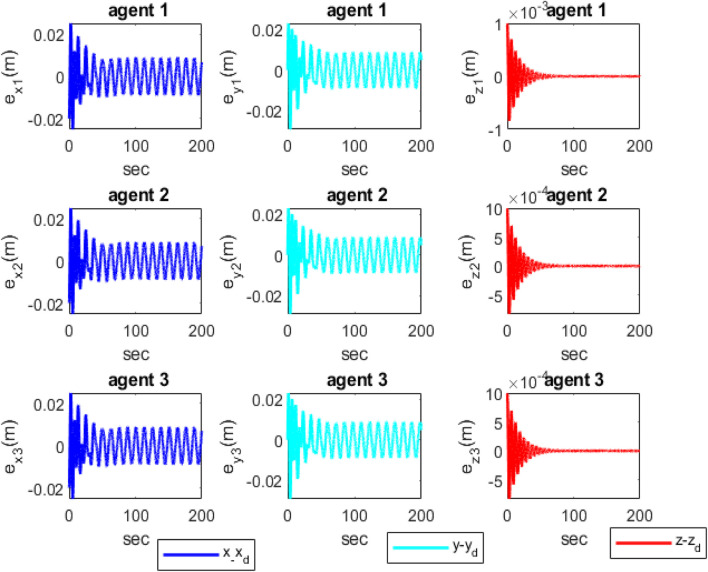
Position tracking errors of the agents in the *x*, *y*, and *z* axes.

**FIGURE 6:**
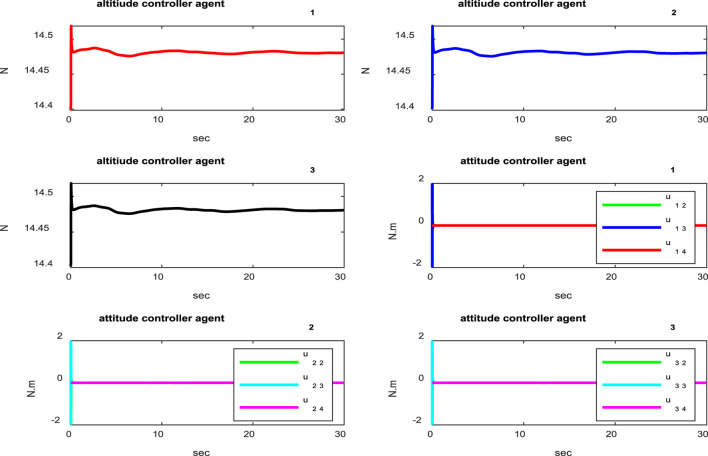
Control signals of the attitude subsystem.

Figure 3 shows the distances between the agents, indicating that the agents are collision-free and maintain distances specified by the reference trajectories between the quadcopters during movement. According to Figure 4, it is clear that the attitude control subsystem is well designed as the states ( ϕ,θ,ψ ) follow the desired trajectories. The position tracking errors of the agents in the *x*, *y*, and *z* axes are depicted in Figure 5, according to which the error is less than 0.03; this shows that the controller is well designed and that the tracking error is acceptable. It is clear from Figure 6 that the value of the control signal $u_{1i},i=1,2,3$ converges approximately to 14.48 N and that the values of the control signals for the attitude subsystem converge to 0 N∙m.

**FIGURE 7:**
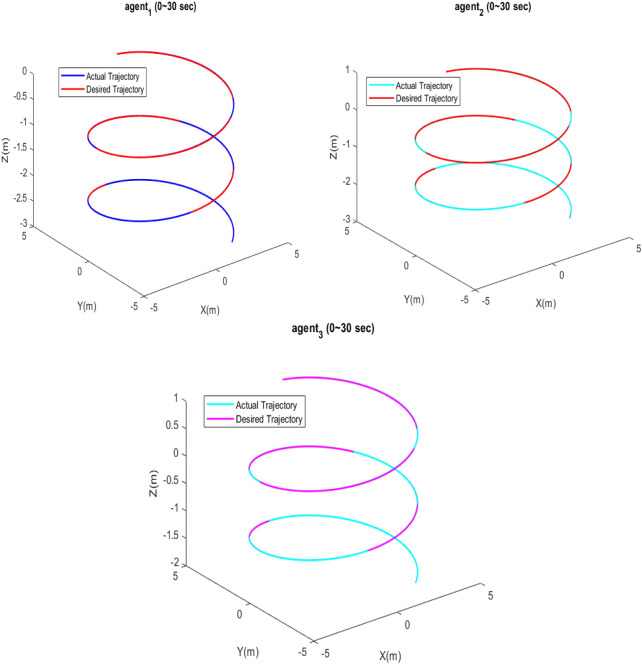
Actual and desired 3D positions of the quadcopters.

**FIGURE 8:**
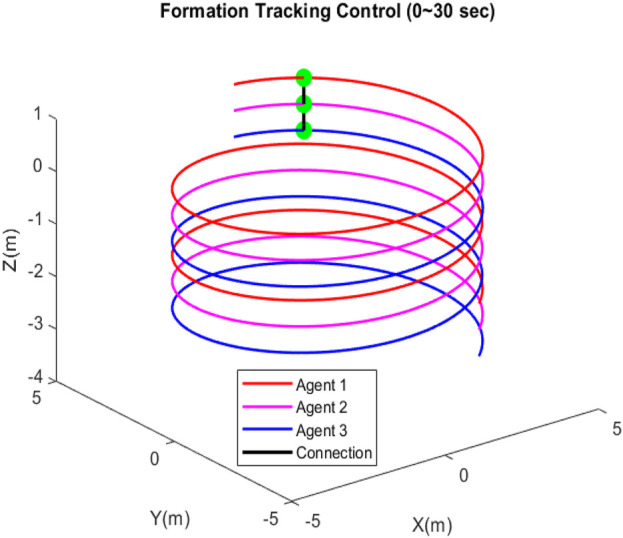
Three-dimensional formation tracking control and formation shape.

**FIGURE 9:**
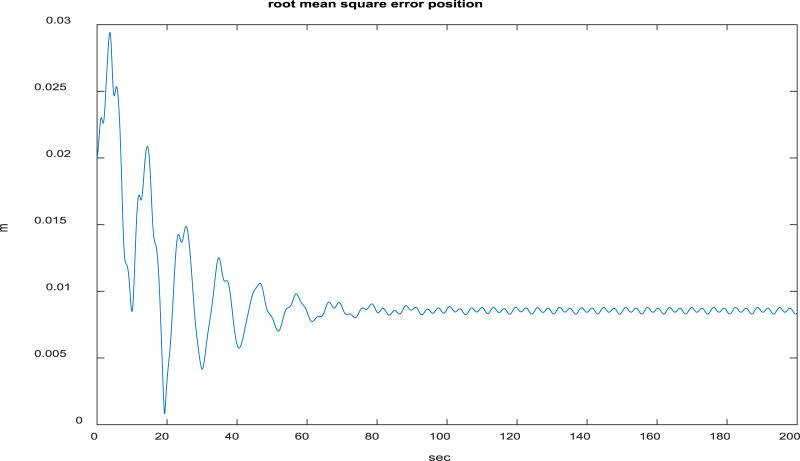
Root mean-squared error (RMSE) between the desired and actual positions of the agents.

Figure 7 depicts that each quadcopter follows its desired path; thus, each quadcopter tracks its desired trajectory successfully during flight. The results of formation tracking as well as the formation shape are depicted in Figure 8; it is obvious from the figure that the quadcopters follow their trajectories in formation without any collisions and that the desired distances between them are maintained. Figure 9 shows that the error value converges to a constant equal to 0.0083, with a settling time of 50 s.

The method proposed in this work is compared with that of Kuriki and Namerikawa (2015) in Table 1, from which it is obvious that the proposed method is significantly superior based on different aspects. The root mean-squared error (RMSE) as well as steady-state error values for the proposed method are considerably lower, and our method is significantly faster. Although the settling time in our method is a bit large, it still outperforms the oscillating behavior of the method proposed by Kuriki and Namerikawa (2015).

**TABLE 1:** Comparison of the proposed method with the system of Kuriki and Namerikawa (2015).

	Settling time (s)	RMSE (m)	Average calculation time (s)	Steady-state error (m)
Proposed method	50	0.0083	0.000053741	0.0083
Kuriki and Namerikawa (2015)	Fluctuating behavior	0.2374	0.005	0.2288

## 7 Conclusion

The purpose of this work was to design a distributed collision-free formation tracking control scheme for multiquadcopter systems using the BLF in a backstepping procedure. The controllers were designed in a hierarchical structure to tackle the underactuated nature of the quadcopter system. Accordingly, the altitude controller was designed first, followed by the translational controller with virtual inputs. The desired Euler angles were then obtained using the virtual control signals and were finally employed to derive the proposed BLF-based attitude control subsystem. Simulations were performed to demonstrate the control objectives designed and achieved herein, including safety (staying in a safe set) and collision avoidance as well as formation tracking control. By adding the uncertainty terms and noise to the dynamics of the system, the controller can be designed such that it meets the control goals when the specified cases occur; this can be considered as a suggestion for future work. Formulating the problem of obstacle avoidance using the BLF is also suggested as a future work.

## Data Availability

The original contributions presented in the study are included in the article/Supplementary material, and any further inquiries may be directed to the corresponding authors.
